# Genome-resolved metagenomics uncovers diversity and functional landscapes of the gastrointestinal epithelium-associated microbiome in cattle

**DOI:** 10.1186/s13059-026-03960-z

**Published:** 2026-01-27

**Authors:** Limei Lin, Xinyi Zheng, Ye Tao, Weiyun Zhu, Le Luo Guan, Shengyong Mao

**Affiliations:** 1 Centre for Ruminant Nutrition and Cleaner Production Innovation, College of Animal Science and Technology, Nanjing Agricultural University, Nanjing, China; 2 Laboratory of Gastrointestinal Microbiology, Nanjing Agricultural University, Nanjing, China; 3 State Key Laboratory of Meat Quality Control and Cultured Meat Development, Nanjing Agricultural University, Nanjing, China; 4 Faculty of Land and Food Systems, The University of British Columbia, Vancouver, Canada; 5 Shanghai BIOZERON Biotechnology Company Ltd, Shanghai, China

## Abstract

**Background:**

The ruminant gastrointestinal epithelium harbors a diverse and functionally critical remains poorly characterized microbial community due to persistent host-derived DNA contamination in metagenomic studies.

**Results:**

We develop Dilute-MetaSeq (dilution-based metagenomic sequencing), a novel, metagenomic workflow integrating gradient dilution with multiple displacement amplification. Dilute-MetaSeq reduces host DNA interference by 52.4-fold and achieves > 90% microbial sequencing efficiency to assess gastrointestinal epithelium-associated microbiome. This enables the construction of the microbial genome atlas of gastrointestinal epithelium (MGA-GE). This comprehensive resource, comprising 1,907 nonredundant prokaryotic and 5,603 viral genomes, reveals extraordinary microbial diversity and novelty, with 41.4% of prokaryotic and 99.9% of viral genomes representing taxonomically unclassified lineages. Spatial profiling identifies the rumen and reticulum as a biodiversity hotspot dominated by epithelium-adapted *Butyrivibrio* and methylotrophic Methanomassiliicoccales, while functional annotation uncovers 1,200 biosynthetic gene clusters (primarily RiPPs and NRPSs) and 1,212 viral auxiliary metabolic genes linked to host metabolism modulation. Pangenome analysis of 987 strains, including a novel *Butyrivibrio* clade with reduced genome sizes, elevated GC content, and butyrate synthesis from amino acid-derived substrates (e.g., glutarate, lysine), highlights metabolic adaptations to the nutrient-scarce epithelial niche compared to digesta-associated microbes.

**Conclusions:**

Collectively, the MGA-GE provides transformative insights into host-microbe-virus interactions and establishes a foundation for developing microbiome-based intervention strategies to enhance ruminant health, agricultural productivity, and bioactive discovery.

**Supplementary Information:**

The online version contains supplementary material available at 10.1186/s13059-026-03960-z.

## Background

Ruminants serve as vital livestock species for producing human-edible products (e.g., milk, meat) and contribute to global agriculture and food security [1]. Unlike other mammals, ruminants have evolved a specialized multi-chambered stomach (rumen, reticulum, omasum, and abomasum) and maintain an obligate mutualistic relationship with their gastrointestinal tract (GIT) microbiota [2]. Diverse microbial consortia in the GIT ecosystem efficiently convert recalcitrant plant biomass into host-accessible nutrients (e.g., volatile fatty acids and microbial protein) and are believed to play a central role in energy conversion and overall metabolic performance [3–5]. Although decades of research on the digesta-associated microbiome of ruminant GITs have provided a fundamental understanding of microbial composition and function [4, 6–8], the microbiota at the gut-lumen interface, particularly the GIT epithelium-colonizing communities, remains largely unexplored. As the primary host-environment exchange interface, epithelial surfaces defend against pathogens while harboring trillions of microorganisms in a complex symbiosis [9–11]. These microbes act as biological gatekeepers, competing with pathogens, facilitating nutrient exchange, and maintaining immune homeostasis [12]. Moreover, the epithelial niche is shaped by the GIT redox gradients, host-derived pressures, and radial biochemical gradients (e.g., pH, oxygen, antimicrobial peptides) [13–15]. Emerging evidence showed that forestomach epithelial microbes reduce carbon flux, whereas hindgut communities utilize host mucopolysaccharides, profoundly influencing local metabolism and host homeostasis [12, 14]. Therefore, profiling the epithelium-associated microbiota in the ruminant GITs is essential to comprehensively elucidate host-microbiome interactions.

Current understanding of the ruminant GIT epithelium associated microbiome (hereafter referred to as the epithelial microbiome) largely stems from taxonomic profiling of microbial communities via targeted marker-gene sequencing (e.g., of 16S/18S rRNA) [16–18]. However, functional insights into microbial metabolic potential, biosynthetic capabilities, and ecological interactions within this specialized niche have been limited in most of these studies due to the absence of comprehensive reference genome frameworks. While advances in metagenomics have enabled the recovery of hundreds of thousands of metagenome-assembled genomes (MAGs) across various microbial ecosystems in a culture-independent manner [6, 19], epithelial microbiome studies face a persistent methodological challenge, where overwhelming host DNA contamination significantly compromises metagenomic analyses [20, 21]. To date, only seven MAGs have been recovered from the rumen epithelium, predominantly representing Campylobacteraceae populations [12]. Overcoming this limitation through innovative approaches represents an urgent priority to unravel the functional ecology of GIT epithelial microbiome. To address this critical gap, we developed a novel metagenomic sequencing method that enhances microbial sequence recovery and enables construction of the microbial genome atlas of the whole gastrointestinal epithelium (MGA-GE) in dairy cattle. The MGA-GE collection comprised 1,907 prokaryotic MAGs and 5,603 viral genomes, revealing remarkable microbial diversity in the GIT epithelium, including a vast reservoir of previously uncharacterized lineages. Functional profiling identified 1,200 biosynthetic gene clusters (BGCs) and 1,212 auxiliary metabolic genes (AMGs), which highlight specialized microbial metabolic adaptations and viral-mediated modulation of host metabolism. Furthermore, comparative genomic analysis of 987 epithelium-associated strains revealed niche-specific variations, nutrient utilization strategies, and metabolic interplay within the GIT epithelial environment. These findings significantly advance our understanding of the microbial ecosystem in ruminant GIT epithelium and offer transformative potentials for strategies to address critical challenges in global agriculture.

## Results

### Construction of the MGA-GE inventory

**Fig. 1:**
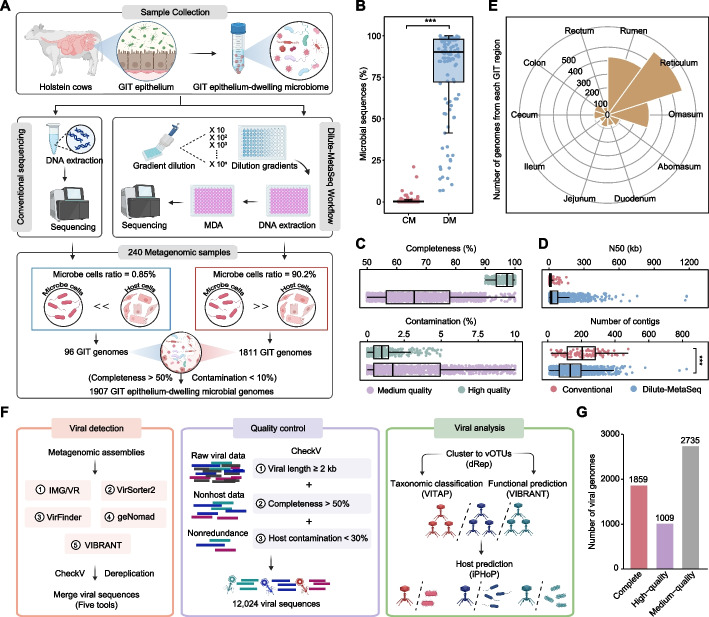
Construction of the Microbial Genome Atlas of Gastrointestinal Epithelium (MGA-GE) in dairy cattle. **A** Pipeline for data acquisition and integration, including conventional metagenomic sequencing and Dilute-MetaSeq Workflow, which was created in BioRender. **B** The proportion of microbial sequencing data obtained using the Dilute-MetaSeq method compared to conventional metagenomic sequencing. CM represents conventional metagenomic sequencing; DM represents Dilute-MetaSeq method. **C** Distribution of completeness and contamination of contigs across the 1,907 MAGs that meet or exceed the medium-quality level. **D** Distribution of N50 and number of contigs across MAGs obtained from Dilute-MetaSeq and conventional sequencing. The distribution is characterized by the minimum, first quartile, median, third quartile, and maximum values. *P < 0.05, **P < 0.01, ***P < 0.001. **E** Number of microbial genomes obtained from each gastrointestinal region. **F** Schematic overview of the viral sequence identification pipeline. **G** The number of complete, high-quality, and medium-quality vOTUs

To address high host sequence contamination in epithelial microbiome studies, we developed a modified metagenomic technique called Dilute-MetaSeq, which combined gradient dilution with multiple displacement amplification (MDA) to enrich microbial DNA (see Methods; Fig. 1A). The Dilute-MetaSeq method significantly improved microbial sequencing rates (median = 90.2%, *n* = 120) compared to conventional metagenomic sequencing (median = 0.85%, *n* = 120) from the entire GIT epithelial samples of dairy cattle, including rumen, reticulum, omasum, abomasum, duodenum, jejunum, ileum, cecum, colon, and rectum (Fig. 1B; two-sided Wilcoxon–Mann–Whitney U-test, *P* < 0.001, Cliff’s δ = 0.998, 95% CI = [0.988, 0.991]). Despite only half the raw sequencing data compared to the conventional method, we obtained 1.4 Tb of high-quality microbial sequences after host sequence depletion, representing a 52.4-fold improvement in microbial recovery (Additional file 1: Table S1 and Additional file 2: Fig. S1A-C; two-sided Wilcoxon–Mann–Whitney U-test, *P* < 0.001, Cliff’s δ = 0.994, 95% CI = [0.976, 0.985]). These results highlight the newly developed Dilute-MetaSeq for overcoming host gene interference, demonstrating its ability to substantially reduce host DNA contamination while significantly enhancing microbial DNA capture.

To comprehensively characterize the epithelial microbiome, we performed single- and co-assembly combined with semi-supervised deep learning binning pipelines (see Methods), obtaining 442 high-quality MAGs (hMAGs: ≥ 90% completeness and ≤ 5% contamination) and 1,465 medium-quality MAGs (mMAGs: ≥ 50% completeness and ≤ 10% contamination) (Additional file 2: Fig. S1D). Among these, 1,811 MAGs were generated by Dilute-MetaSeq (hMAGs = 416; mMAGs = 1,395), while 96 were produced through conventional metagenomic sequencing (hMAGs = 26; mMAGs = 70) (Fig. 1C; Additional file 2: Fig. S1D). The MAGs generated using Dilute-MetaSeq contained significantly fewer assembly fragments per genome than those obtained with conventional methods (Fig. 1D; phylogenetic generalized least squares, PGLS: Δ = − 50.1, 95% CI = [− 73.0, − 27.2], *P* < 0.001). Furthermore, Dilute-MetaSeq produced 58 MAGs containing rRNA operons (23S, 16S, and 5S genes) and tRNA genes, whereas conventional sequencing yielded only 2 such MAGs (Additional file 1: Table S2). These results revealed that Dilute-MetaSeq not only recovered more MAGs but also achieved higher genomic quality from the GIT epithelium, providing a foundation for genome-resolved metagenomic exploration. MAGs were distributed across all 10 GIT regions, with the highest recovery from the reticulum (*n* = 578), rumen (*n* = 428), and omasum (*n* = 307), while hindgut compartments yielded 62–104 MAGs each (Additional file 1: Table S2; Fig. 1E). These MAGs displayed substantial genomic diversity, with genome sizes ranging from 0.63 to 9.4 Mb (median = 2.7 Mb) and GC contents varying between 23.4% and 69.2% (median = 49.7%) (Additional file 1: Table S2). Quality assessment showed an average completeness of 74.4% (median = 72.6%) and low contamination levels (average = 2.5%; median = 1.4%). We also recovered viral genomes from the GIT epithelial microbiome using a newly developed hybrid computational pipeline integrating five different analysis methods (see Methods; Fig. 1F). After dereplication and prophage excision, we identified 12,024 viral genomes (> 50% completeness, < 30% host contamination, and > 2 kb length; Fig. 1F), which clustered into 5,603 nonredundant viral operational taxonomic units (vOTUs) at 95% average nucleotide identity (ANI) over 85% alignment coverage (Fig. 1F). Among these, 33.2% vOTUs were classified as complete genomes, 18% as high-quality, and 48.8% as medium-quality (Fig. 1G; Additional file 1: Table S3). Additionally, over 66.5% of vOTUs originated from the forestomach (*n* = 3,725), followed by the ileum (*n* = 526), jejunum (*n* = 469), cecum (*n* = 223), and colon (*n* = 218), with the duodenum contributing the smallest number (*n* = 82) (Additional file 2: Fig. S1E). These results revealed a previously overlooked but substantial viral component in the GIT epithelium. Collectively, the 1,907 MAGs and 5,603 vOTUs derived from the GIT epithelium of dairy cattle constitute the MGA-GE collection, providing a comprehensive genomic resource for investigating host-microbiome-virus interactions in epithelial ecosystems.

### Taxonomic classification and community structure of the MGA-GE

**Fig. 2:**
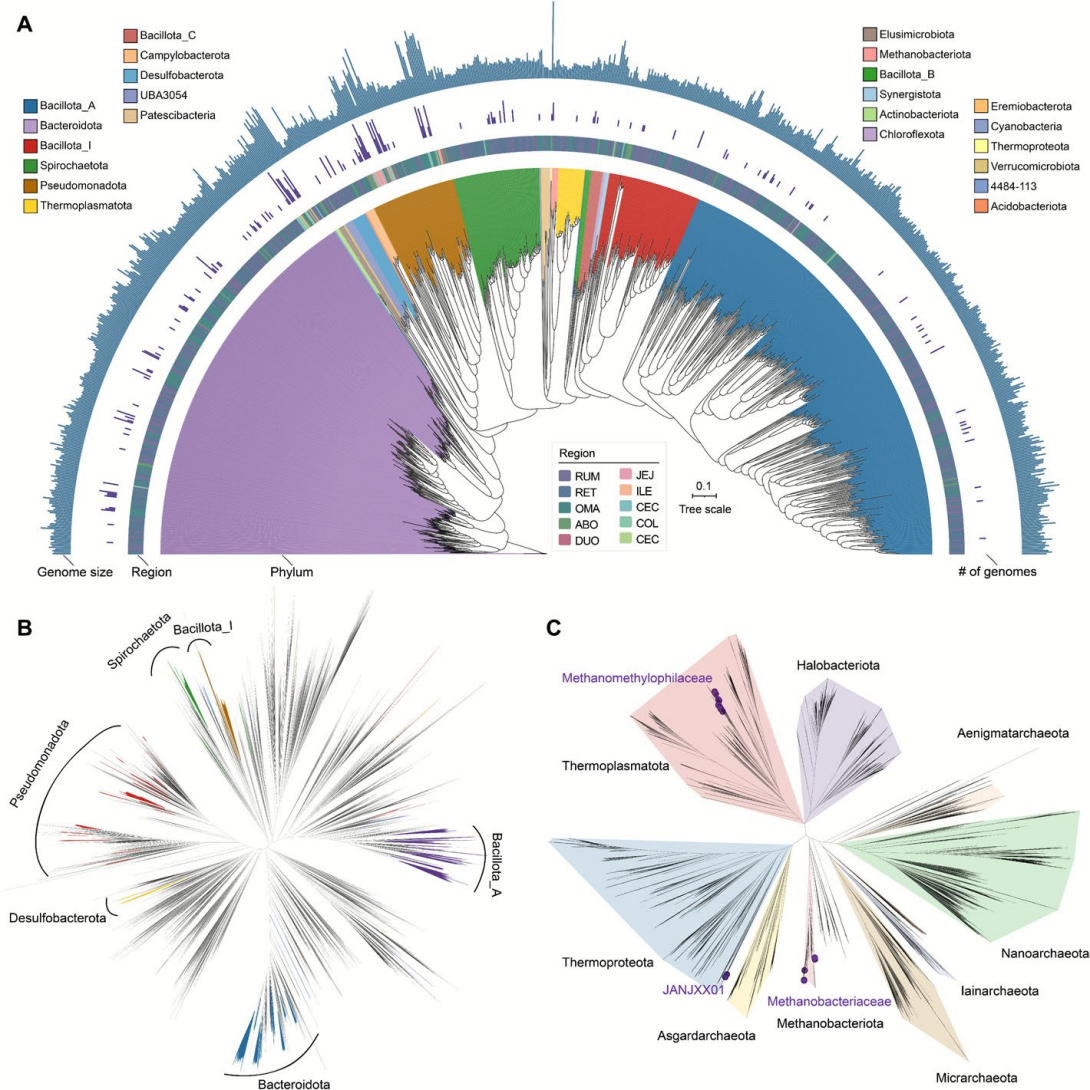
Phylogenetic relationships and taxonomic classification of MAGs from the gastrointestinal epithelium in dairy cattle. **A** Phylogenetic tree of 987 strain-level genomes of the DCGEM catalogue. The maximum-likelihood tree is constructed using PhyloPhlAn and visualized using the online iTOL tool. Branches are shaded with color to highlight phylum-level affiliations. The heat map represents the strains from different GIT regions. The inner bar ring indicates the number of genomes represented within each strain. The outside layer of the bar graph represents the genome size of each genome. RUM, rumen; RET, reticulum; OMA, omasum; ABO, abomasum; DUO, duodenum; JEJ, jejunum; ILE, ileum; CEC, cecum; COL, colon; REC, rectum. **B** Unrooted phylogenomic analysis of 1,870 bacterial genomes derived from this study and representative strains in GTDB (Release 220), with major phyla highlighted by branch color. **C** Unrooted phylogenomic analysis of 37 archaeal genomes obtained from this study and GTDB representatives. Strains belonging to the phyla are distinguished by distinct background colors. The 37 archaeal genomes derived from this study are marked with solid purple circles

To explore the composition of the MGA-GE, we taxonomically classified the prokaryotic MAGs using the GTDB database [22], revealing 23 phyla, 29 classes, 59 orders, 92 families, and 202 genera. The community was dominated by Bacteroidota (34.8%), Pseudomonadota (26.6%), and Bacillota_A (21.5%), with notable contributions from Spirochaetota (5.1%), Bacillota_I (4.6%), Desulfobacterota (2%), Thermoplasmatota (1.2%), Methanobacteriota (0.6%), and Bacillota_C (0.6%). Moreover, Thermoproteota, Eremiobacterota, Cyanobacteriota, Bacillota, Chloroflexota, and Verrucomicrobiota were identified as rare phyla, collectively accounting for only 0.1% of the total genomes. Among genera, *Cryptobacteroides* (7.4%), *Prevotella* (4.7%), and *Butyrivibrio* (4.6%) were most abundant, followed by *Treponema*_D (4.2%), *Egerieousia* (3.6%), UBA636 (3.4%), *Eubacterium*_B (2.7%), and *Bulleidia* (2.6%) (Additional file 1: Table S2). While Dilute-MetaSeq is optimized for genome recovery and its compositional accuracy remains to be established, the recovered genomes nevertheless correspond closely to epithelial-enriched taxa reported in 16S rRNA gene sequencing and metatranscriptomic studies [17, 23, 24]. Notably, 789 genomes lacked species-level classification, mostly from the Bacillota_A (phylum) and Lachnospiraceae (family), suggesting the presence of potential novel lineages within these two common taxonomic groups. Using a strain-level threshold (> 99% ANI), we identified 987 distinct strains (Fig. 2A), with 62.8% (*n* = 620) representing novel taxa unclassifiable at the species level, revealing substantial unexplored microbial diversity in the epithelial niche. Phylogenomic analysis integrating GTDB classifications showed these novel lineages were phylogenetically closest to functionally important genera: *Butyrivibrio* [25] (butyrate production), *Treponema*_D [26] (oxygen scavenging), and *Desulfobulbus* [27] (sulfate reduction) (Fig. 2B). This phylogenetic proximity suggests their unclassified relatives may play analogous roles in host energy metabolism and epithelial maintenance. Additionally, we detected 35 archaeal genomes forming two distinct clades: 23 Methanomethylophilaceae and 12 Methanobacteriaceae members (Fig. 2C), indicating two specialized archaeal communities in the epithelial microenvironment. These findings also highlight archaeal potential contributions in host-microbiome interactions through their metabolic significance [28]. Strain clustering revealed the highest diversity of unique strains in the rumen, reticulum, and omasum, contrasting with fewer than 10 strains per compartment in the small and large intestines (Fig. 2A). These findings suggest that the compartmentalized stomach of ruminants harbors distinct microbial lineages, where unique physicochemical gradients across the rumen, reticulum, and omasum may drive evolutionary divergence and niche specialization [29].

Our alignment-based analysis against ICTV reference standards revealed substantial viral novelty, with 49.1% (*n* = 2,750) of vOTUs showing no significant similarity to classified viruses, representing previously uncharacterized viral clades. Among classified vOTUs, the class Caudoviricetes dominated (92.6%; Additional file 1: Table S4), yet exhibited remarkable taxonomic novelty that > 99.9% remained unclassified below the class level. The taxonomic novelty of all vOTUs extended to finer resolutions, including 90.8% unclassified at the family, 99.8% at the genus levels, and 99.9% at the species level, revealing a vast diversity of niche-adapted viral lineages. Through host prediction, we associated 23.3% of vOTUs (*n* = 1,305) with prokaryotic hosts, predominantly infecting Pseudomonadota (*n* = 578), Bacillota_A (*n* = 418), and Bacteroidota (*n* = 405), with *Comamonas* being the most frequently targeted genus (Additional file 2: Fig. S2). The remaining vOTUs likely infect uncultured or taxonomically uncharacterized prokaryotes. Notably, 14.8% (*n* = 193) of host-associated vOTUs demonstrated cross-genera infectivity (Additional file 1: Table S5), suggesting their potential role in facilitating genetic exchange and microbial adaptation within the epithelium niche.

### Phylogenomic diversity and niche specialization of the MGA-GE

**Fig. 3:**
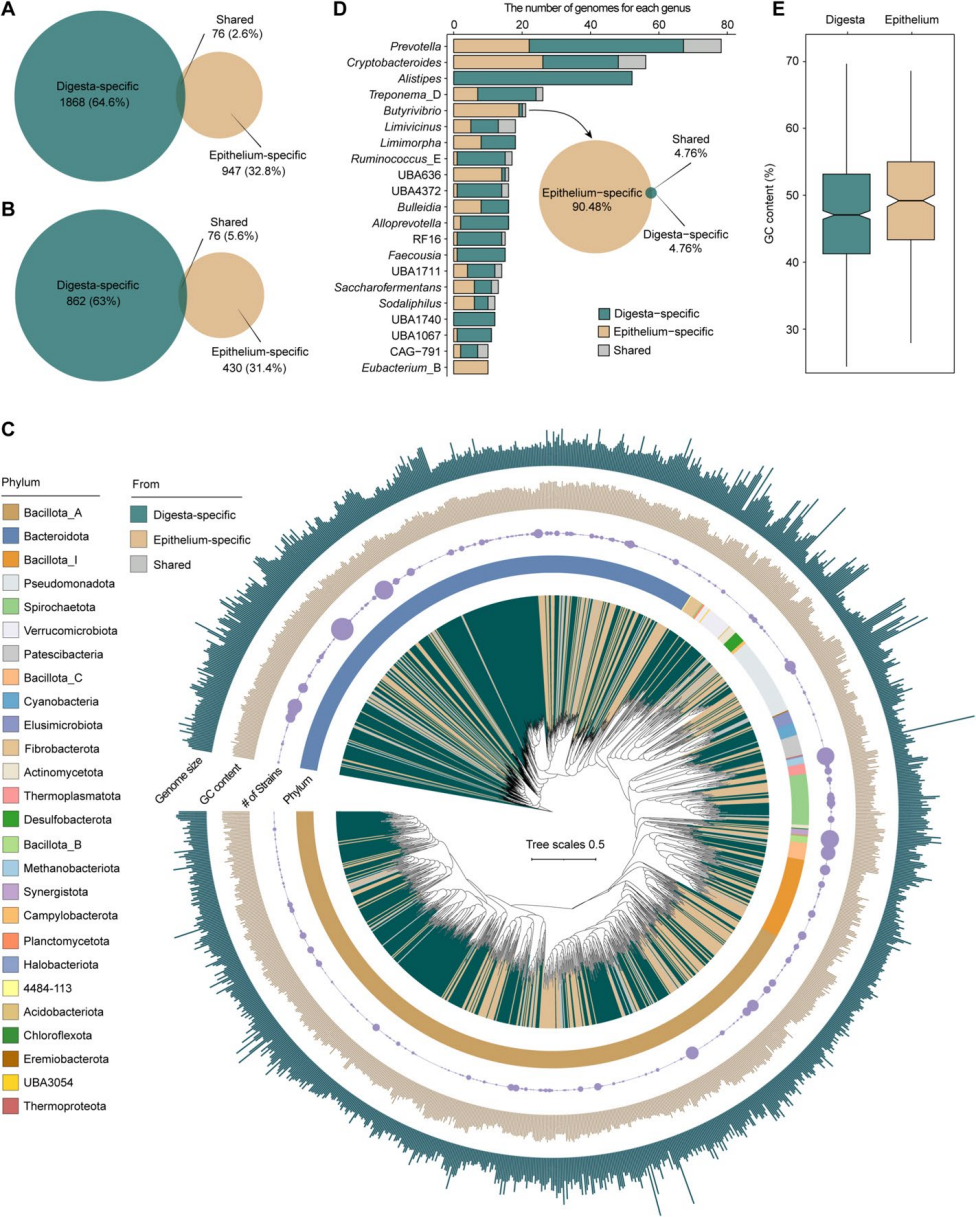
Comparative genomic analysis of microbial diversity in the gastrointestinal epithelium and digesta. **A** The number of epithelium-specific, digesta-specific, and shared strains based on 99% whole-genome ANI. **B** The count of epithelium-specific, digesta-specific, and shared species derived from 1,368 prokaryotic species, based on 95% whole-genome ANI. **C** A genome-wide phylogenetic tree of 1,368 prokaryotic species obtained from the gastrointestinal epithelium and digesta. Branches are shaded with color to highlight the source of species, including epithelium-specific, digesta-specific, and shared in both. The heat map represents phylum-level affiliations. The circle indicates the number of strains corresponding to each species. The inside layer of the bar graph represents the GC content of each genome. The outside layer of the bar graph represents the genome size of each genome. **D** Taxonomic distribution at the genus level of the 430 epithelium-specific species. The vertical axis represents the number of genomes for each genus. **E** Comparison of genomic GC content between epithelium-specific and digesta-specific species

Building upon the taxonomic characterization, we next assessed the phylogenomic diversity and ecological specialization of the MGA-GE by comparing it with 1,904 MAGs derived from dairy cattle digesta-associated microbiomes (Fig. 3A) [7]. Species-level clustering (ANI > 95%) identified 1,368 distinct genomic clusters, revealing pronounced niche partitioning: 31.4% were epithelium-specific, 63% digesta-specific, and only 5.6% shared between niches (Fig. 3B). Epithelium-specific species spanned 22 phyla, 87 families, and 183 genera, with dominant genera being *Cryptobacteroides* (6%), *Prevotella* (5.1%), *Butyrivibrio* (4.4%), UBA636 (3.3%), and *Eubacterium*_B (2.3%) (Fig. 3C, D; Additional file 1: Table S6). In contrast, digesta-specific species were enriched in polysaccharide-degrading taxa, including *Alistipes* (6%), *Prevotella* (5.2%), *Cryptobacteroides* (2.6%), and *Treponema*_D (2%) (Fig. 3D). Comparative genomic analysis indicated that epithelium-specific species exhibited numerically higher GC content than digesta-specific species; but this difference did not reach statistical significance (Fig. 3E; phylogenetic generalized least squares, PGLS: Δ = 0.41%, 95% CI = [−0.39, 1.22], *P* = 0.312). Notably, 67.2% of these epithelium-specific species lacked a known taxonomic classification (Additional file 1: Table S6), revealing a substantial reservoir of uncharacterized microbial diversity. These results indicate the existence of a highly specialized microbial community adapted to the epithelial environment. Subsequent analysis identified 19 *Butyrivibrio* species distinctly adapted to the epithelium of the rumen, reticulum, and omasum (Additional file 2: Fig. S3 and Additional file 1: Table S6). Moreover, spatial profiling revealed heterogeneity across the GIT epithelium. Species from *Desulfobulbus*, *Campylobacter*_B, RGIG9985, *Ottowia*, and *Prevotella* predominantly dominated the forestomach epithelium, and a previously unclassified *Helicobacter* species was detected in the abomasal epithelium. Phylogenomic analysis against 59 reference *Helicobacter* genomes placed this lineage close to taxa isolated from the gastric mucosa of humans and monogastric animals (e.g., *Helicobacter pylori*) (Additional file 2: Fig. S4), indicating the need for further investigation. By contrast, *Treponema*_F species were associated with the hindgut epithelium (Additional file 2: Fig. S3 and Additional file 1: Table S6). These findings suggest that the stratified structure of the ruminant stomach supports distinct microbial communities, with the upper compartments serving as biodiversity hotspots likely shaped by localized physicochemical gradients and nutrient fluxes [7]. In addition to bacterial taxa, 60% of archaeal-specific species belonged to the methylotrophic Methanomassiliicoccales [30], with forestomach enrichment suggesting niche adaptation (Additional file 2: Fig. S3 and Additional file 1: Table S6). These methanogens can metabolize trimethylamine, which is absorbed through the epithelium and converted to trimethylamine N-oxide (TMAO) in the liver, a process linked to increased vascular diseases risk in humans [31, 32]. These observations suggest that Methanomassiliicoccales may contribute to reducing systemic TMAO accumulation, thereby potentially influencing host health, although further experimental validation is needed to substantiate this hypothesis [28]. Furthermore, 67.9% of epithelium-specific species were represented by singleton MAGs, reflecting their rarity in the GIT epithelium microbiome. Collectively, these findings revealed a distinct microbial consortium at the GIT epithelium, dominated by unclassified and novel lineages.

To evaluate the diversity of epithelium-associated viruses, we performed a comparative analysis of viral genomes between the GIT epithelium and digesta. Strikingly, only 50.9% of epithelial viruses could be taxonomically classified compared to 81.2% in the digesta (Additional file 1: Table S7), underscoring the extensive uncharacterized diversity of epithelial viral populations. Taxonomic classification revealed a predominance of Caudoviricetes (99.5%) among digesta vOTUs, while epithelial viruses displayed greater diversity, with significant representation of Malgrandaviricetes (4.5%), Arfiviricetes (1.4%), and Faserviricetes (1.2%), suggested a potential ecological relevance of these viral groups within the epithelial niche. Host prediction analysis further demonstrated that digesta vOTUs were predominantly predicted to infect Bacillota_A (*n* = 1,028) and Bacteroidota (*n* = 887), with only 11.2% exhibiting multi-genera infectivity (Additional file 2: Fig. S5 and Additional file 1: Table S8). In contrast, epithelial viruses exhibited preferential targeting of Pseudomonadota and significantly higher multi-genera infectivity (14.8%), indicating an enhanced potential to facilitate cross-genus microbial interactions within the epithelial niche. Such interactions may facilitate virus-mediated horizontal gene transfer and enhance community resilience under nutrient stress, thereby accelerating microbial adaptation to the epithelial niche [33]. These findings offered a critical avenue for exploring viral-mediated microbial evolution and its implications for host health in sustainable livestock systems.

### Diverse secondary metabolite biosynthetic potential in the GIT epithelium-associated microbiome

**Fig. 4:**
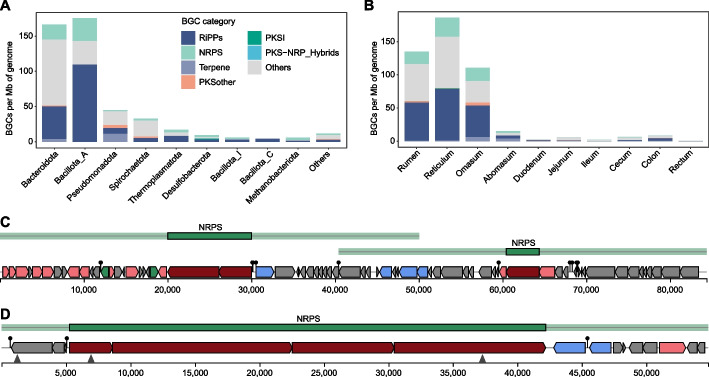
Biosynthetic Gene Clusters (BGCs) identified in the MGA-GE dataset. **A** Distribution of normalized BGC counts across dominant phyla. The stacked bar plots show the density of BGCs (BGCs per Mb of genome) within each phylum, with colors indicating distinct BGC categories. **B** Distribution of normalized BGC counts across different gastrointestinal regions, with stacked bars indicating the relative contribution of each BGC category. **C** The largest single BGC region, identified in a reticulum-derived bacterium putatively classified within the phylum Pseudomonadota and genus *Serratia*. **D** A 40-kb non-ribosomal peptide synthetase (NRPS) cluster from a jejunum-derived *Pseudomonas*_E strain, predicted to encode an antimicrobial peptide

The GIT epithelium harbors a host-regulated microbiome that thrives in a complex environment with specialized metabolic substrates. To investigate microbial secondary metabolite production in this niche, we analyzed BGCs, which are genomic regions that encode the enzymatic machinery responsible for secondary metabolite synthesis in MGA-GE MAGs using antiSMASH [34]. A total of 1,200 putative BGCs were predicted from 642 MAGs spanning 19 bacterial phyla, with 75.1% (*n* = 901) exhibiting no homology to entries in the MIBiG database, indicating substantial biosynthetic novelty. These BGCs were classified into seven categories, with ribosomally synthesized and post-translationally modified peptide (RiPP; 38.3%, *n* = 460) being the most abundant (Fig. 4A; Additional file 2: Fig. S6 and Additional file 1: Table S9). RiPPs displayed broad phylogenetic distribution across 13 prokaryotic phyla, reflecting their ecological significance in the GIT epithelial. We further identified 190 non-ribosomal peptide synthetase (NRPS; 15.8%), 53 terpene (4.4%), 23 other polyketide synthase (PKS; 1.9%), 3 PKS I (0.3%), and 2 PKS-NRPS hybrid (0.2%) gene clusters, distributed across 10, 2, 4, 2, and 2 phyla, respectively (Additional file 2: Fig. S6). Bacillota_A and Bacteroidota collectively accounted for 71.9% of the BGC counts after normalization by genome size (BGCs per Mb; Fig. 4A), while Pseudomonadota exhibited the greatest diversity of BGC types (including terpene, RiPPs, NRPSs, other PKSs, and PKS-NRPS hybrids). Interestingly, Desulfobacterota uniquely harbored all PKS I gene clusters, implying phylogenetic specialization in epithelial polyketide biosynthesis. Normalized BGC counts were predominantly observed in the forestomach epithelium (90.8% of total), with RiPPs and NRPSs showing the highest number in these regions (Fig. 4B), suggesting these regions function as a hotspot for antimicrobial/signaling compound production. In contrast, terpene gene clusters showed wider distribution across lower gut regions (Fig. 4B), likely supporting distinct ecological functions.

Using our standardized pipeline, we comparatively analyzed BGCs encoded by 1,904 digesta-derived MAGs versus the MGA-GE collection. Epithelium-associated MAGs exhibited 1.3-fold greater BGC density per genome than digesta-associated MAGs (Additional file 1: Table S10), revealing enhanced individual biosynthetic potential in the GIT epithelial communities. Strikingly, the MGA-GE contained several BGC classes absent in digesta-associated MAGs, including those encoding T3PKS, lanthipeptide-class-I, proteusin, NRPS-NRP-metallophore, and resorcinol-arylpolyene (Additional file 1: Table S10), indicating niche-specific metabolic adaptations to epithelial conditions. Notably, 142 epithelial BGCs (11.8%) exceeded 30 kb, including an 84.4-kb mega-cluster in a reticulum-derived *Serratia* (Pseudomonadota) containing multiple NRPS modules (Fig. 4C) and a 40-kb jejunum-derived *Pseudomonas*_E NRPS cluster predicted to produce antimicrobial peptides (Fig. 4D), suggesting capacity for complex metabolite synthesis. These expansive BGCs likely encode novel bioactive compounds, positioning the GIT epithelium as a valuable source for uncharacterized microbial chemistry that demands heterologous expression and synthetic biology approaches for functional characterization.

### Microbial metabolic specialization and functional plasticity in the GIT epithelium

**Fig. 5:**
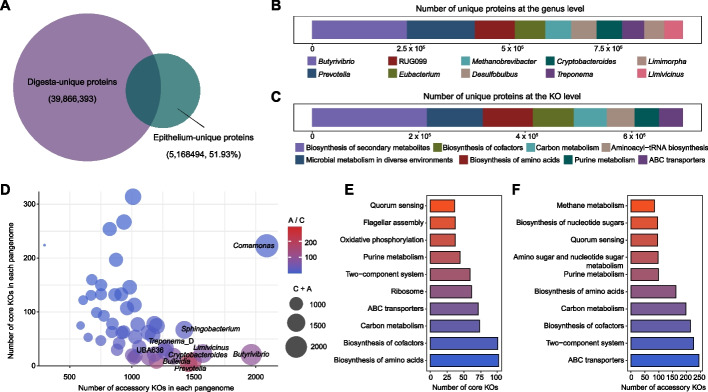
Functional landscape and intra-population genomic variation analyses within the gastrointestinal epithelium microbiome. **A** The number and proportion of digesta-unique and epithelium-unique proteins. **B** Taxonomic annotation of epithelium-unique proteins at the genus level. **C** KEGG annotation of epithelium-unique proteins. **D** The number of core and accessory KOs in each pangenome across the 49 pangenomes representing 687 strains. **E** KEGG pathways associated with core KOs across the 49 pangenomes. **F** KEGG pathways associated with accessory KOs across the 49 pangenomes. A, represents the number of accessory KOs; C, represents the number of core KOs; A/C represents the ratio of the number of accessory KOs to the number of core KOs; C + A represents the total number of core and accessory KOs

We further constructed metabolic networks and compiled a catalog of 9,952,861 nonredundant microbial gene clusters, with 40.4% lacking functional annotation in the eggNOG database, revealing substantial uncharacterized functional potential. Comparative analysis with 45,886,195 digesta-derived genes identified 51.9% of genes as epithelium-specific (95% identify threshold; Fig. 5A). Taxonomic analysis showed *Butyrivibrio* (4.7%), *Prevotella* (3.3%), and RUG099 (2%) as top contributors of unique genes, followed by *Eubacterium* (1.5%), *Methanobrevibacter* (1.3%), *Desulfobulbus* (1.3%), *Cryptobacteroides* (1.3%), and *Treponema* (1.1%) (Fig. 5B). Beyond secondary metabolite biosynthesis, these genes primarily functioned in amino acids/cofactors biosynthesis, aminoacyl-tRNA production, carbon metabolism, and ABC transporters (Fig. 5C). Among 1,212 viral-encoded AMGs representing 116 KEGG orthologous families, functional annotation revealed predominant roles in amino acid metabolism (51.1%), cofactors/vitamins metabolism (14.3%), energy metabolism (11.7%), and carbohydrate metabolism (10.1%) (Additional file 2: Fig. S7A). Enrichment analysis further highlighted their strong association with cysteine/methionine and sulfur metabolism pathways (Additional file 2: Fig. S7B). These findings supported viral modulation of host metabolic flexibility, consistent with prior evidence that viruses potentially modulate amino acid metabolism to enhance energy production while contributing to sulfur cycling under nutrient limitation [35, 36]. The antioxidant-linked nature of these pathways underscores their ecological significance [37]. The AMG repertoire, particularly its enrichment in amino acid, sulfur, and energy metabolism, positions viruses as key regulators of epithelial ecosystems. By augmenting host metabolic flexibility and antioxidant capacity, these AMGs likely promote microbial resilience, representing a critical adaptive mechanism for ecosystem stability. Collectively, these results suggest that the GIT epithelium harbors specialized metabolic networks, with viruses potentially contributing to community functionality through enhanced antioxidant defenses and energy provisioning in nutrient-limited environment.

To characterize functional diversity within the MGA-GE, we constructed 49 pangenomes representing 987 strains (≥ 5 genomes each; Fig. 5D), identifying 1,621 core genes (shared by > 90% strains) and 4,741 accessory genes (specific to one or a few strains). Core genes primarily mediated essential cellular processes, including amino acids/cofactors biosynthesis and carbon metabolism (Fig. 5E), while accessory genes were enriched for environmental adaptation traits, particularly secondary metabolites biosynthesis (Fig. 5F). This dichotomy highlights how accessory gene pools enhance metabolic plasticity and ecological resilience, reinforcing the epithelial microbiome’s exceptional BGC diversity and its role in niche specialization. Notably, core/accessory gene ratios exhibited genus-specific patterns. The rumen epithelium-dominant genus *Butyrivibrio* showed marked accessory gene expansion coupled with core gene reduction (Fig. 5D), suggesting strong selection for metabolic versatility in adapting to dynamic epithelial conditions. In addition, 80% of epithelium-associated *Butyrivibrio* MAGs encoded pilus assembly proteins (Additional file 1: Table S11), indicating an enhanced capacity for epithelial attachment and persistence. Such genetic architecture likely facilitates substrate flexibility and microenvironmental adaptation, enhancing *Butyrivibrio*’s ecological dominance in the GIT epithelium.

### New *Butyrivibrio* strains revealed divergent substrate utilization preferences and butyrate production

**Fig. 6:**
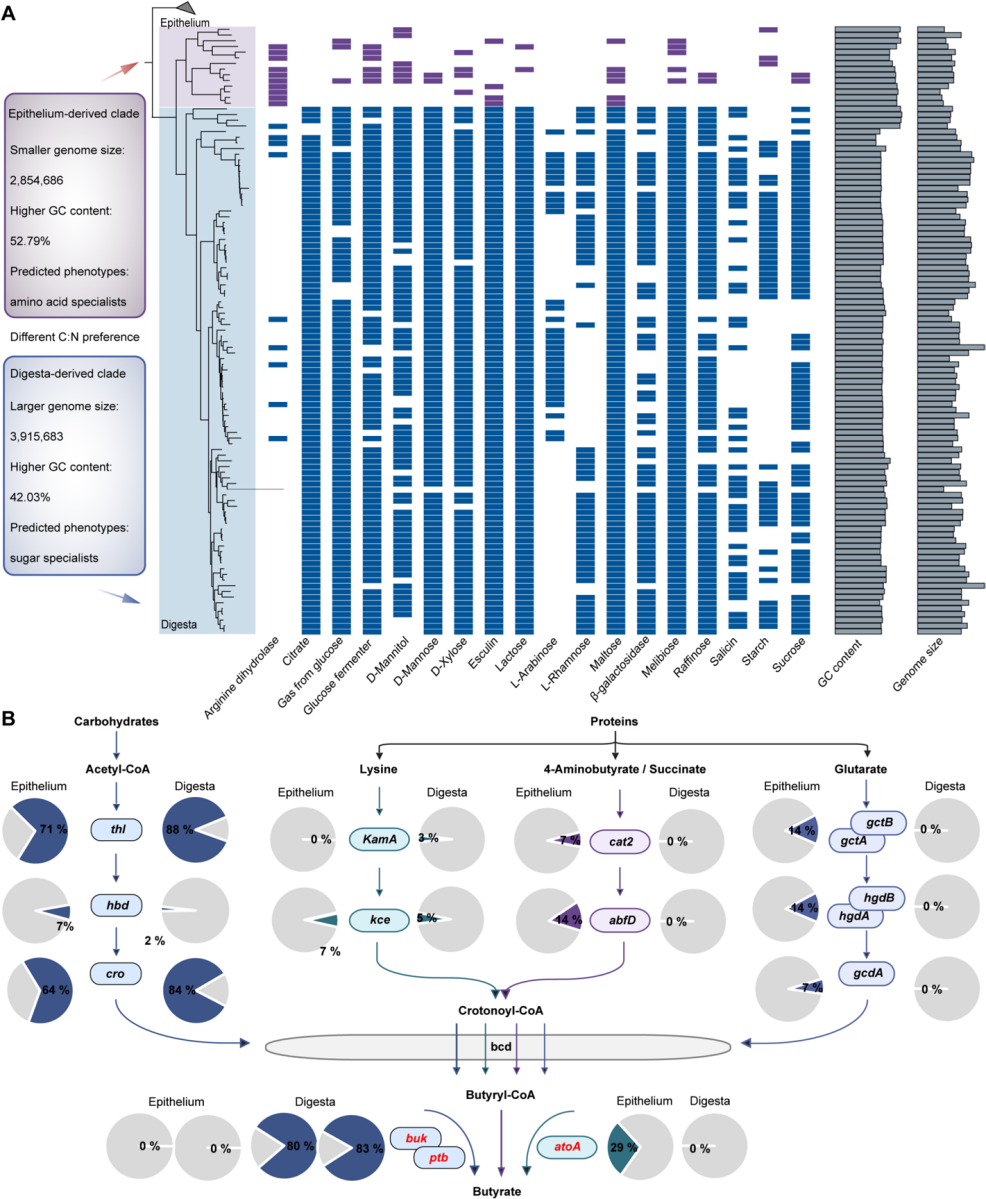
Divergent metabolic preferences of novel *Butyrivibrio* clades in the rumen epithelium.** A** Phylogenetic relationship, genomic GC content, genome size, and substrate-utilization profiles of 107 *Butyrivibrio* strains, highlighting an epithelium-associated clade (purple) and a digesta-associated clade (blue). Colored squares indicate the presence of genes enabling utilization of corresponding substrates. **B** Differences in butyrate production pathways between epithelium-associated and digesta-associated *Butyrivibrio* clades. Four different pathways for butyrate synthesis and their corresponding genes are displayed, with major substrates indicated. Terminal genes are highlighted in red. The pie chart represents the proportion of genomes capable of encoding these genes within the epithelium-associated clade or digesta-associated clade. *buk*, butyrate kinase; *ptb*, phosphate butyryltransferase; *ato*, butyryl-CoA:acetoacetate CoA transferase (α subunit); thl, acetyl-CoA acetyltransferase (thiolase); *hbd*, 3-hydroxybutyryl-CoA dehydrogenase; *cro*, crotonase; *kamA*, lysine-2,3-aminomutase; *kce*, 3-keto-5-aminohexanoate cleavage enzyme; *cat2*, 4-hydroxybutyrate CoA-transferase; *abfD*, 4-hydroxybutyryl-CoA dehydratase; *gct*, glutaconate CoA-transferase (α, β subunits); *hgd*, 2-hydroxyglutaryl-CoA dehydratase (α, β subunits); *gcd*, glutaconyl-CoA decarboxylase (α subunit); *bcd*, butyryl-CoA dehydrogenase

*Butyrivibrio* spp. play a pivotal role in maintaining rumen homeostasis and host health through their specialized butyrate-producing capabilities, yet the functions of epithelium-associated *Butyrivibrio* spp. remain unclear. We thus conducted a comprehensive analysis integrating 54 publicly available *Butyrivibrio* isolates from ruminal digesta, 36 ruminal digesta-derived MAGs [4, 6, 38–46], and 17 epithelium-associated *Butyrivibrio* MAGs from the rumen, enabling a robust comparative study of their genomic and ecological traits (Additional file 1: Table S12). Phylogenomic reconstruction of these 107 strains revealed a distinct epithelium-associated clade (comprising 14 taxonomically unclassified strains) that was phylogenetically separated from digesta-associated genomes (Fig. 6A). This epithelial clade exhibited numerically smaller genome sizes (2.9 ± 0.14 Mb) compared to digesta-associated genomes (3.9 ± 0.07 Mb); however, after accounting for phylogenetic relatedness, this difference was not statistically significant (PGLS: Δ = − 0.30 Mb, 95% CI = [− 1.61, 1.00], *P* = 0.681). In contrast, GC content was significantly higher in epithelial genomes (PGLS: Δ = 10.6%, 95% CI = [6.97, 14.3], *P* = 0.021) (Fig. 6A). Since smaller genomes are often associated with nutrient-limited environments [47], we speculated that the reduced genome size in epithelial *Butyrivibrio* strains reflects an adaptive response to the nutrient-scarce epithelial niche, particularly under limited carbon availability (e.g., lignocellulose and starch). Notably, nutrient availability also influences bacterial GC content [48]. Phenotypic prediction across all 107 *Butyrivibrio* genomes, based on genomic variation, showed that most digesta-associated genomes primarily relied on sugar fermentation and lacked the ability to utilize amino acids for growth (Fig. 6A). These strains were predicted to metabolize diverse substrates, including D-xylose, lactose, L-arabinose, L-rhamnose, maltose, D-mannose, D-mannitol, melibiose, esculin hydrolysis, β-galactosidase, raffinose, salicin, starch, and sucrose (Fig. 6A). In contrast, most epithelium-associated MAGs lacked sugar utilization capacity but exhibited metabolic activity for amino acids, particularly arginine (Fig. 6A). This pattern aligned with prior observations in other microbes, where low-GC-content genomes preferentially encode sugar metabolism pathways, while high-GC-content genomes favor nitrogen-rich amino acid metabolism [48–50]. These findings demonstrated clear ecological specialization, as epithelium-associated *Butyrivibrio* strains have evolved adaptations for amino acid metabolism.

We further hypothesized that butyrate biosynthesis in epithelium-associated *Butyrivibrio* spp. may fundamentally differ from that of digesta-associated strains. Although *Butyrivibrio* species typically employ the glycolytic acetyl-CoA pathway for butyrate production [51, 52], the observed sugar metabolism deficiency in epithelial strains may suggest alternative pathways. Indeed, all epithelium-associated *Butyrivibrio* genomes lacked genes encoding the key terminal enzymes (phosphate butyryltransferase, *ptb*, and butyrate kinase, *buk*) of the acetyl-CoA pathway, whereas nearly all digesta-associated genomes retained the complete pathway (Fig. 6B; Additional file 2: Fig. S8). Careful inspection of the genomic loci further confirmed the reliable absence of *ptb* and *buk* in epithelial strains (Additional file 2: Fig. S9). Moreover, several epithelium-associated *Butyrivibrio* MAGs encoded genes (e.g., *atoA*; butyryl-CoA:acetoacetate CoA transferase) for alternative butyrate synthesis pathways using amino acids (including glutarate, 4-aminobutyrate, and lysine), which were absent in digesta-associated genomes (Fig. 6B). Collectively, these findings suggested that rumen epithelium-associated *Butyrivibrio* strains occupy a unique substrate niche by employing distinct butyrate synthesis pathways. The shift in epithelial *Butyrivibrio* strains toward amino acid-derived butyrate production, bypassing the traditional acetyl-CoA pathway, represents a profound metabolic adaptation to the nutrient-scarce epithelial niche. This strategy likely optimizes energy yield from limited carbon sources while supporting host epithelial cell proliferation via butyrate, a key mediator. This divergence provides avenues for manipulating *Butyrivibrio* spp. metabolism to boost butyrate availability, potentially improving ruminant energy efficiency and resilience amid dietary shifts. These insights advance our understanding of microbial nutrient utilization, metabolic adaptation, and ecological interactions, establishing a transformative foundation for future host-microbiome research to address global agricultural challenges.

## Discussion

Dilute-MetaSeq enabled high-yield recovery of epithelial genomes across the bovine GIT, overcoming host DNA interference and generating the MGA-GE resource of 1,907 prokaryotic genomes and 5,603 vOTUs. Through genome-resolved comparison with paired digesta datasets, we revealed a spatial organizational gradient from lumen to the epithelium [12, 29]. Among 1,368 species-level genomic clusters, 31.4% were epithelium-specific, 63.0% were digesta-specific, and only 5.6% were shared, indicating strong niche partitioning. Even within genera detected in both compartments, such as *Prevotella*, distinct species compositions were identified, illustrating clear species-level turnover at the host-environment interface.

Microbial distribution was shaped collectively by oxygen availability [53, 54]. The lumen is strictly anoxic and dominated by carbohydrate-fermenting specialists that generate volatile fatty acids. In contrast, the epithelial surface is mildly oxygenated due to host oxygen leakage and supports a community adapted to host-derived solutes within a physiologically hypoxic boundary environment. Consistent with adaptation to micro-oxygen conditions, Pseudomonadota accounted for 26.6% of epithelial genomes, higher than their abundance in the digesta [7]. On the reducing side, *Desulfobulbus* spp. encoded sulfate respiration pathways, likely resulting in H₂S production that may quench reactive oxygen species as a collateral benefit of energy metabolism [55–57]. Notably, our genomic data revealed the presence of pilus assembly proteins in *Desulfobulbus* (Additional file 1: Table S11), suggesting an enhanced ability for epithelial adhesion [58]. This feature helps stabilize their colonization in microaerobic niches where localized H₂S production could mitigate oxidative stress near the host interface. These findings support a model of functional complementarity between the two compartments, with the epithelial microbiota relying on microaerobic and sulfate-based mechanisms, while the lumen is dominated by obligate fermenters.

Within this ecological framework, epithelial metabolism also appears strategically organized around exogenous electron donors derived from host leakage, particularly amino acids and other nitrogenous compounds, in contrast to the lumen where microbes rely mainly on carbon substrates. Spatial profiling further revealed high strain-level diversity along the forestomach epithelia, indicative of strong niche-specific selection pressures. A key example of such functional specialization is illustrated by *Butyrivibrio*: epithelial-associated genomes were numerically smaller in size (though not statistically significant after phylogenetic correction), exhibited higher in GC content, and lacked *ptb* and *buk*, the essential enzymes of the canonical acetyl-CoA pathway [51, 52]. Instead, they encoded alternative pathways for butyrate synthesis from amino acid precursors such as glutarate, 4-aminobutyrate, and lysine. This metabolic reprogramming shifts butyrate production from canonical carbohydrate-based pathways toward amino acid-derived substrates, resulting in a carbohydrate-independent source of butyrate at the epithelial surface. Given that butyrate serves as a primary energy source for epithelial cells, these alternative synthetic routes provide a direct mechanism for sustaining epithelial turnover and absorptive function, particularly under conditions of carbohydrate limitation. Furthermore, the identified presence of pilus assembly proteins in these epithelial *Butyrivibrio* strains suggests enhanced adhesive capacity [58], facilitating persistent colonization and localized delivery of butyrate to host tissues, thereby reinforcing microbial ecological persistence. In our dataset, we also observed enrichment of *Campylobacter* spp. along the forestomach epithelia, and given that a subset of epithelial Campylobacteraceae strains has previously been shown to utilize acetate [12], the phylogenetic clustering of our MGA-GE *Campylobacter* genomes with these lineages (Additional file 2: Fig. S10) further supports the notion that the epithelial niche selects for taxa with specialized carbon-utilization capacities. Collectively, these adaptive traits illustrate a sophisticated mechanistic link between microbial metabolic specialization and the maintenance of host epithelial barrier function.

Beyond core metabolic functions, the epithelial microbiome was found to harbor a significantly higher density and diversity of BGCs compared to the digesta community, with 75.1% of these BGCs representing novel classes. Among these, several were rare or absent from microbial genomes in the digesta, including those encoding lanthipeptides and proteusins (associated with antimicrobial activity and biofilm formation) [59, 60], NRPS-derived metallophores (implicated in iron and zinc acquisition under nutrient-limited mucosal conditions) [61], as well as aryl polyene and resorcinol biosynthesis pathways (linked to oxidative stress resistance) [62]. These traits align with the challenges of a host-shaped epithelial environment that demands sustained colonization, efficient micronutrient acquisition, and protection against redox fluctuations. An additional functional layer was contributed by viruses, which not only exhibited high genomic novelty within the epithelial niche but also demonstrated significantly higher cross-genus infectivity than viral populations in the digesta. Many of these viruses carried AMGs enriched in functions related to amino acid, sulfur, and energy metabolism. Beyond their role as horizontal gene transfer vectors, these viruses may also mediate top-down control of epithelial-associated microbial populations, thereby actively shaping community composition and functional dynamics at the host interface. The concurrence findings support the hypothesis that the epithelial niche serves as a reservoir for microbial evolution and genetic exchange [63], fostering adaptive traits that may shape rumen community function and ecological resilience.

These genomic findings enable us to propose several testable mechanisms through which the epithelial microbiome may influence host physiology. Among these potential mechanisms, the production of butyrate from amino acids by epithelial *Butyrivibrio* spp. is most directly supported by our genomic data (presence of *atoA*, absence of *ptb*/*buk*), providing a testable model for how these microbes support epithelial energetics. The proposed antioxidant buffering and TMAO reduction mechanisms, while consistent with observed gene inventories, require direct experimental validation. Concurrently, the production of butyrate from amino acids by epithelial-associated *Butyrivibrio* spp. could provide a carbohydrate-independent energy source directly supporting epithelial cell turnover and absorption function. Furthermore, the enrichment of methylotrophic methanogens indicates a possible reduction in trimethylamine availability, thereby attenuating host synthesis of TMAO, a metabolite linked to metabolic dysregulation [28, 31, 32]. Collectively, these microbiota-host interactions may enhance epithelial barrier resilience, modulate immune responses, and influence systemic metabolic homeostasis. Although these remain hypotheses awaiting experimental validation, they point to compelling functional interactions.

The implications of our findings extend well beyond ruminants. Host-regulated epithelial surfaces are a common feature in non-ruminant herbivores and monogastric animals, where microbial communities face similar selective pressures, including nutrient scarcity, oxidative stress, and immune activity [13–15]. Methodologically, Dilute-MetaSeq effectively overcomes host DNA contamination and can be broadly applied to establish high-quality genome-resolved epithelial microbial catalogs across diverse host species. Biologically, the detection of *Helicobacter* spp. in the abomasum epithelium mirrors the prevalence of microaerophilic lineages in the gastric mucosa of monogastric species, suggesting possible implications for mucosal health, including potential roles in low-grade inflammation, gastric adaptation, or opportunistic colonization in cattle. Although pathogenicity cannot be inferred from genomic data alone, the presence of these lineages underscores the need for future work to determine their functional significance and potential health impacts. The recurrence of oxygen-scavenging and epithelium-adherent taxa underscores functional conservation across vertebrates. This phylogenetic functional convergence suggests that certain microbial lineages represent evolutionarily conserved solutions for colonizing gastric and intestinal mucosal surfaces. Consequently, the resulting MGA-GE dataset may thus serve as a valuable resource for identifying universal principles governing the organization of host-associated microbial communities.

## Conclusions

The development of the Dilute-MetaSeq methodology and the establishment of the MGA-GE represented a significant advancement in our understanding dairy cattle GIT epithelial ecosystems, overcoming longstanding host DNA contamination barriers to reveal unprecedented microbial diversity comprising 1,907 prokaryotic genomes and 5,603 vOTUs, with a substantial proportion of novel lineages. Spatial comparison identified the forestomach epithelium as a biodiversity hotspot dominated by specialized taxa, including *Butyrivibrio* and Methanomassiliicoccales. Notably, rumen epithelial *Butyrivibrio* strains exhibited a unique amino acid-derived butyrate synthesis pathway, distinct from those in digesta-derived counterparts, highlighting a key metabolic adaptation to the nutrient-limited epithelial niche. Furthermore, cataloging of prokaryotic BGCs and viral AMGs revealed remarkable biosynthetic potential and virus-mediated metabolic reprogramming capacities within this niche. The discovery of epithelium-specific lineages with reduced genomes and alternative metabolic strategies underscores how nutrient constraints drive evolutionary innovation at the host interface. These collective findings establish the MGA-GE as an indispensable resource for advancing gastrointestinal health, feed efficiency, and bioactive compound discovery, while creating new opportunities to investigate host-microbe-virus interactions through targeted culturing and multi-omics approaches that translate genomic insights into functional applications for sustainable agriculture.

## Methods

### Animals and sample collection

Twelve healthy Holstein dairy cows with comparable body weight (651 ± 54 kg) and lactation stage (233 ± 16 days in milk) were selected for this study. Following humane euthanasia prior to morning feeding, complete necropsy was performed with immediate dissection of GIT segments, including the rumen, reticulum, omasum, abomasum, duodenum, jejunum, ileum, cecum, colon, and rectum. Epithelial tissue samples were collected from three forestomach regions (rumen, reticulum, and omasum), while mucosal layers were harvested from the remaining seven GIT regions (abomasum, duodenum, jejunum, ileum, cecum, colon, and rectum). All GIT epithelial specimens underwent sequential processing: 1) thorough rinsing with ice-cold phosphate-buffered saline (PBS), 2) mechanical scraping using sterile glass slides under aseptic conditions, and 3) flash-freezing in liquid nitrogen for preservation prior to DNA extraction.

### DNA extraction and conventional metagenomic sequencing

Total genomic DNA was isolated from 120 epithelial samples using the DNeasy PowerSoil Pro Kit (Qiagen, Hilden, Germany) following the manufacturer’s protocol [12]. DNA integrity was verified through 0.8% agarose gel electrophoresis, while purity and concentration were quantified spectrophotometrically using the Nanodrop ND-1000 (Thermo Fisher Scientific, Waltman, MA, USA). Metagenomic libraries were prepared from high-quality DNA extracts (insert size of 350 bp) using the TruSeq DNA PCR-Free Library Preparation Kit (Illumina, San Diego, CA, USA) according to the manufacturer’s instructions, with subsequent paired-end sequencing (2 × 150 bp) performed on the NovaSeq 6000 platform (Illumina, San Diego, CA, USA).

### DNA extraction and Dilute-MetaSeq metagenomic sequencing

To minimize host DNA contamination and enhance microbial genome recovery from GIT epithelium, we implemented the Dilute-MetaSeq protocol across all 120 GIT epithelial samples. This approach combined gradient dilution with MDA [64] for microbial DNA enrichment. Briefly, 1–2 g of epithelial tissue was homogenized in 5–10 mL of ice-cold 1 × PBS (5 mL per gram of tissue), vortexed for 5 min, and centrifuged at 500 × g for 5 min at 4 °C to pellet debris. The supernatant was transferred to fresh tubes and centrifuged at 17,000 × *g* for 5 min at 4 °C to collect microbial cells, and the resulting pellet was used for DNA extraction with the DNeasy PowerSoil Pro Kit (Qiagen, Hilden, Germany). To determine the optimal dilution factor, serial dilutions (1:10 to 1:10^6^) of microbial suspensions from rumen and colon epithelial samples were tested, and host DNA contamination was quantified by qPCR targeting the *Bos taurus* actin beta (*ACTB*) gene (F-primer: CATCGGCAATGAGCGGTTCC; R-primer: ACCGTGTTGGCGTAGAGGTC). Each 20 μL reaction contained 2 μL of extracted DNA, 5 pmol of each primer, and 1 × SYBR Green qPCR Master Mix (QK Platinum SYBR Green Master Mix, catalogue A57156; Thermo Fisher Scientific), and reactions were run on the SLAN-96P Real-Time PCR System (Hongshitech, China) under the following conditions: 95 °C for 10 min, followed by 40 cycles of 95 °C for 30 s, 60 °C for 30 s, and 72 °C for 30 s, with fluorescence recorded at the end of each cycle; a final melting curve analysis was performed at 95 °C for 1 min, 55 °C for 30 s, and 95 °C for 30 s. In both rumen and colon tests, dilutions at 1:1000 consistently yielded Ct values > 25, indicating negligible host DNA (Additional file 2: Fig. S11). Accordingly, this dilution was adopted for all 120 epithelial samples. DNA from these optimized dilutions was subsequently amplified using the REPLI-g Single Cell Kit (Qiagen, Cat. no. 150343), generating > 1 μg of DNA per sample for library preparation and sequencing under the same conditions as conventional metagenomic workflows. Negative controls (ddH₂O, no-template MDA) included in parallel exhibited no detectable amplification (Additional file 2: Fig. S12), thereby confirming the absence of cross-contamination. Additionally, MDA may introduce known biases, such as preferential amplification of small circular genomes and the formation of chimeric products [65]. To mitigate these artifacts, we applied several downstream steps: short, low-confidence contigs were removed based on length thresholds; coverage- and composition-aware binning reduced the influence of contigs with chimeric profiles; and CheckM/dRep refinement excluded bins with structural inconsistencies or inflated contamination.

### Quality control, sequence assembly, taxonomic classification, and functional annotation

Illumina sequencing data from both conventional and Dilute-MetaSeq metagenomic analyses of the GIT epithelium-associated microbiome underwent quality control using Fastp [66] (v.0.20.1; parameters: -detect_adapter_for_pe -q 25 −5 −3 -l 100) for adapter trimming and low-quality read removal. Quality-controlled reads were mapped to the *Bos taurus* reference genome (GCF_002263795.2) using BWA-MEM [67] (v.0.7.17), and host-mapped reads with an alignment score ≥ 30 were removed (Additional file 1: Table S1). We additionally performed k-mer–based validation of host depletion using sourmash [68] (v.4.9.4), which confirmed host signals were effectively absent. Host reference genomes were sketched (k = 31, 51; scaled = 1000) and compared against the clean reads using containment analysis. High-quality reads from each of the 240 metagenomic samples were de novo assembled individually using metaSPAdes [69] (v.3.15.4). For the conventional metagenomic sequencing data, open reading frames (ORFs) were predicted using Prodigal [70] (v.2.6.3; parameter: -p meta), yielding 15.89 million (M) ORFs, of which 79.12% were complete. These ORFs were clustered with CD-HIT [71] (v.4.8.1) to generate a nonredundant microbial gene catalog comprising 9,952,861 genes. This gene catalog was taxonomically and functionally annotated using DIAMOND [72] (v.2.0.13; *E*-values < 1e^−5^) for BLASTP searches against the NCBI-NR and eggNOG [73] databases, and KofamScan [74] (v.1.1.0) for profiling against the Kyoto Encyclopedia of Genes and Genomes (KEGG) [75] database. High-quality reads from each sample were aligned to the gene catalog using BWA-MEM [67] (v.0.7.17) and relative abundance was quantified as transcripts per million (TPM) [76] (alignment criteria: length ≥ 50 bp and sequence identity > 95%). The relative abundance of taxa, KOs, and COGs were summarized based on the annotated gene abundances [77].

### Binning and refinement of MAGs

To improve microbial diversity characterization in the GIT epithelium, we performed metagenomic binning on both conventional and Dilute-MetaSeq datasets. In addition to single-sample assemblies, we co-assembled high-quality reads from regionally matched samples using MEGAHIT [78] (v.1.2.9; parameters: -min-contig-len 100 -presets meta-sensitive -prune-depth 1). Contigs from single and co-assemblies were mapped to paired-end reads using BWA-MEM [67] (v.0.7.17), and resulting SAM files were coordinate-sorted with SAMtools [79] (v.1.9) to generate BAM files. Contigs < 2,500 bp were excluded, unless those between 1,000 to 2,500 bp constituted < 5% of total base pairs, in which case the threshold was lowered to 1,000 bp [80]. Filtered contigs were binned independently using SemiBin [80] (v.1.5.1; single_easy_bin model), which leverages a semi-supervised learning for deep siamese neural network with reference genome data. Bin quality was assessed via CheckM (v.1.2.1; parameter: lineage_wf), and genome sizes were corrected and estimated based on their completeness and contamination [81]. From 240 metagenomes, we obtained 1,907 MAGs with > 50% completeness and < 10% contamination, including 442 high-quality MAGs (> 90% completeness and < 5% contamination). These were dereplicated into 987 strain-level MAGs (99% ANI) using dRep (v.3.4.5; parameters: -pa 0.95 -sa 0.99 -cm larger) [82]. To identify epithelium-specific strains/species, we clustered 2,891 prokaryotic genomes (987 epithelium-derived MAGs and 1,904 digesta-derived MAGs [7]) at 99% (strain-level) and 95% (species-level) ANI. The relative abundance of genome was calculated using CoverM [83] (v.0.6.1; parameters: -min-read-percent-identity 0.95 -min-read-aligned-percent 0.75 -trim-min 0.10 -trim-max 0.90 -m tpm -proper-pairs-only).

### Phylogenetic, taxonomic, and functional analysis of MAGs

Genome taxonomy was assigned using the GTDB-Tk [22] (v.2.3.2) classify workflow with the GTDB release r220. A maximum-likelihood phylogenomic tree was constructed with PhyloPhlAn [84] (v.3.0.67). The best tree was refined using RAxML and visualized with iTOL [85] (v.6; https://itol.embl.de). Gene calling and annotation were performed using Prokka [86] (v.1.14.6; default parameters). Functional classification and metabolic reconstruction were conducted by querying predicted protein sequences against the eggNOG database (BLASTP-based searched with DIAMOND [72], v.2.0.13; *E*-value < 1e^−5^), the KEGG database (annotated with KofamScan [74] v.1.1.0; only high-confidence assignments marked with asterisks were retained), and HMMER [87] (v.3.3.2) for additional domain-based analysis.

### Pangenome analysis and inter-population genomic diversity

Pangenome analyses were performed on 49 genera comprising 687 gastrointestinal epithelium-associated prokaryotic strains (≥ 5 genomes per genus), each represented by at least five genomes. We defined core KEGG Orthologs (KOs) as those present in ≥ 90% of genomes, while accessory KOs were those unique to individual or small groups of genomes. For *Butyrivibrio* specifically, we analyzed 107 strains total: 17 from rumen epithelium (this study), 36 from ruminal digesta MAGs [4, 6, 38–46], and 54 publicly available isolates (Additional file 1: Table S12). Pairwise ANI comparisons were conducted using BLASTN (v.2.13.0) for ANIb calculations and MUMmer [88] (v.3.0) for ANIm calculations. Both analyses were implemented through pyANI [89] (v.0.2.12). Phenotypic traits were predicted for 107 *Butyrivibrio* strains using Traitar [90] (v.1.1.12). We retained only annotations where predictions from both the basic phypat classifier and the enhanced phypat + PGL classifiers (incorporating evolutionary phenotype gain/loss information) were concordant.

### Secondary metabolite BGCs prediction of prokaryotic genomes

BGCs were systematically identified from MAG-derived contigs (≥ 5 kb) using antiSMASH [34] (v.7.1.0) with stringent parameters for accurate gene boundary prediction. All predicted BGCs underwent functional categorization via BiG-SCAPE [91] (v.1.1.8) into seven canonical classes: PKS I, PKSother, NRPS, RiPPs, terpenes, PKS-NRP hybrids, and other atypical clusters. Novelty assessment was performed by cross-referencing all BGCs against the Minimum Information about a Biosynthetic Gene Cluster database (MIBiG) [92] (v.3.1) through antiSMASH’s built-in comparison module (parameter: -cb-knownclusters), with sequence identity thresholds and synteny analysis used to distinguish known from potentially novel clusters.

### Identification and quality control of viral genomes

To maximize viral sequence identification from the dairy cattle GIT epithelium, we implemented an integrated five-tool pipeline combining complementary detection approaches: searching IMG/VR [93] (v.4), VirSorter2 [94] (v.2.2.4), VirFinder [95] (v.1.1), and geNomad [96] (v.1.11.0), and VIBRANT [97] (v.1.2.1). Each tool employs distinct algorithms to enhance the sensitivity and specificity of viral contig identification from metagenomic assemblies. First method aligned contigs against the IMG/VR (v.4) reference database using BLASTn (v.2.13.0; parameters: -max_target_seqs 5, -evalue 1e-5), with best hits filtered and annotated to ensure high-confidence viral predictions. VirSorter2 software implemented customized classifiers to detect diverse viral types, filtering out sequences containing fewer than two viral marker genes and those identified as partial. VirFinder software utilized a machine learning model to identify viral k-mer frequency patterns, retaining hits with scores ≥ 0.7 and *P* ≤ 0.05 for high specificity. geNomad software applied a hybrid framework combining alignment-free and gene-based models, executed in end-to-end mode with default database cleanup for precise viral contig identification. VIBRANT software employed a hybrid approach of machine learning and protein similarity, running with default settings to recover viral sequences based on functional gene content rather than sequence features alone. Viral sequences identified by these five tools were aggregated into a comprehensive sequence pool. Consensus viral sequences identified by ≥ 2 tools were consolidated and subjected to rigorous quality control using CheckV [98] (v.1.0.3; end-to-end mode) for completeness assessment, followed by dereplication via the aniclust.py script (parameters: -min_ani 100, -min_tcov 100, -min_qcov 0). Only sequences meeting stringent quality criteria were retained: 1) completeness > 50%, 2) host contamination < 30%, and 3) contig length ≥ 2 kb. This pipeline generated a final set of 12,024 high-confidence viral sequences from the epithelium and 13,819 from the digesta of the dairy cattle GIT.

### Virus clustering, taxonomic classification, functional annotation, and host prediction

For viral genome analysis, we performed comprehensive characterization through four key steps. Viral sequences were clustered into species-level viral Operational Taxonomic Units (vOTUs) using dRep [82] (v.3.4.5; parameters: –ignoreGenomeQuality -pa 0.8 -sa 0.95 -nc 0.85 -comW 0 -conW 0 -strW 0 -N50W 0 -sizeW 1 -centW 0 -cm larger), ensuring precise and reliable delineation. Taxonomic classification of each vOTU was conducted using VITAP [99] (v.1.7), which incorporates an extensive viral reference database and utilizes both sequence similarity and phylogenetic indicators to assign accurate species-level annotations. Functional annotation of vOTUs was performed with VIBRANT [97] (v.1.2.1), employing a hybrid methodology that combines machine learning with protein similarity searches to identify AMGs and other functionally important viral elements. Using default parameters, a total of 1,212 putative AMGs were initially recovered. To ensure stringent curation, we conducted additional manual inspection using three criteria: (i) the AMG had to be located on a viral contig containing hallmark genes, as defined by VOG annotations with keywords including portal, terminase, spike, capsid, sheath, tail, coat, virion, lysin, holin, base plate, lysozyme, head, or structural; (ii) within the ± 10 gene neighborhood of the AMG, at least one viral hallmark gene must be present; (iii) contigs containing bacterial multi-copy core genes (e.g., 16S rRNA) were excluded. Applying these criteria resulted in 37.4% of the initially detected AMGs being retained as high-confidence candidates (Additional file 1: Table S13 and Additional file 2: Fig. S13). Finally, host-virus associations were predicted using iPHoP [100] (v.1.3.3) with default parameters, which leverages a carefully curated database of virus-host interactions to predict potential hosts with high confidence.

### Statistical analyses

Read-level comparisons between Dilute-MetaSeq and conventional metagenomic sequencing (microbial sequencing rate, host sequencing rate, and the number of high-quality microbial reads) were conducted using two-sided Wilcoxon–Mann–Whitney U-test with Benjamini–Hochberg false discovery rate (BH–FDR) correction, and we additionally reported Cliff’s δ with 95% bootstrap confidence intervals. Genome-resolved traits (GC content, genome size, N50 contig length, and number of contigs) were analyzed using phylogenetic generalized least squares (PGLS), reporting the group contrast as Δ (defined by the model contrast; e.g., Epithelium – Digesta) with 95% confidence intervals and BH–FDR-adjusted *P*. A maximum-likelihood phylogenomic tree was constructed with PhyloPhlAn [84] (v.3.0.67).

## Supplementary Information

Supplementary Material 1. Contains Tables S1-S13.

Supplementary Material 2. Contains Figs. S1-S13.

## Data Availability

All raw sequencing reads have been submitted to the National Center for Biotechnology Information (NCBI) at Sequence Read Archive under BioProject accession No. PRJNA1260059 [101]. All genomes utilized in this study have been deposited in Figshare [102] (https://doi.org/10.6084/m9.figshare.28988897). A documented computational workflow, including all steps from raw read processing to MAG reconstruction, software version information, and usage instructions, is available at GitHub repository [103] (https://github.com/LLMmay/MGA-GE, GPL-3.0 license) and has been archived with a DOI in Zenodo [104] (https://doi.org/10.5281/zenodo.18224675). R scripts used for figure generation are also provided in the same repository.
