# Supplementary material for: Genome-resolved metagenomics uncovers diversity and functional landscapes of the gastrointestinal epithelium-associated microbiome in cattle
Source: Genome Biol. 2026 Jan 27;27:44. doi: 10.1186/s13059-026-03960-z (PMC12918008; doi:10.1186/s13059-026-03960-z)
Supplement: Supplementary file 2 — Supplementary Material 2. Contains Figs. S1-S13. [file 13059_2026_3960_MOESM2_ESM.docx]

**Fig. S1 A** The proportion of host sequencing data obtained using the Dilute-MetaSeq method compared to conventional metagenomic sequencing. Host sequences % (two-sided Wilcoxon–Mann–Whitney U-test, *P* < 0.001, Cliff’s δ = −0.998, 95% CI [−0.991, −0.988]; CM = 120, DM = 120). CM represents conventional metagenomic sequencing; DM represents Dilute-MetaSeq method. **B** Per-region distributions of host sequences (%). Individual sample points are overlaid on box plots for each gastrointestinal region. Paired CM vs. DM comparisons (two-sided Wilcoxon signed-rank, BH-FDR across Regions) are annotated in the panel; full statistics are provided in Supplementary Table S1. **C** Per-region distributions of microbial data (log10 Gb). RUM, rumen; RET, reticulum; OMA, omasum; ABO, abomasum; DUO, duodenum; JEJ, jejunum; ILE, ileum; CEC, cecum; COL, colon; REC, rectum. **D** Genome quality of the 1,907 MAGs. The MAGs are displayed according to the genome completeness and contamination rates. Genome numbers are color-coded based on their sources, with CM represented in green and DM in purple. **E** Number of viral genomes obtained from each gastrointestinal region. **P* < 0.05, ***P* < 0.01, ****P* < 0.001.

**Fig. S2** Host prediction for vOTUs derived from the gastrointestinal epithelium of dairy cattle. Host predictions are presented for a curated subset of vOTUs, selected based on a minimum of 10 assignments to a single genus, with taxonomic classifications spanning domain, phylum, class, order, family, and genus levels.

**Fig. S3** Phylogenetic tree of 430 epithelium-specific species. A maximum-likelihood phylogenetic tree was constructed using PhyloPhlAn and visualized with the iTOL online tool. Branch colors denote order-level taxonomic affiliations. A heatmap adjacent to the tree indicates the presence of each strain across different GIT regions: rumen, reticulum, omasum, abomasum, duodenum, jejunum, ileum, cecum, colon, and rectum. Genus-level classifications are shown as strip charts.

**Fig. S4** Maximum-likelihood phylogenetic tree of 60 *Helicobacter* species. The tree was constructed using PhyloPhlAn and visualized with the iTOL online tool. The red pentagram indicates the *Helicobacter* species from the abomasum epithelium in this study, while the other 59 species represent reference genomes retrieved from NCBI.

**Fig. S5** Host prediction for vOTUs derived from the gastrointestinal digesta of dairy cattle. Host predictions are presented for a curated subset of vOTUs, selected based on a minimum of 10 assignments to a single genus, with taxonomic classifications spanning domain, phylum, class, order, family, and genus level.

**Fig. S6** The sunburst chart illustrates the global composition of BGC types across all genomes in the dataset: inner segments represent seven major BGC classes with relative percentage, while outer segments provide subclassification of “Others” (aryl polyene, betalactone, phosphonate) and RiPPs (RRE-containing, ranthipeptide) with absolute counts. Smaller classes such as PKS I and PKS-NRP_Hybrids are labeled externally for clarity.

**Fig. S7 A** Distribution of 1,212 viral-encoded AMGs across major metabolic categories, annotated using KEGG pathways. **B** Enrichment of these 1,212 viral-encoded AMGs within specific KEGG pathways.

**Fig. S8** Phylogenetic distribution and presence/absence of butyrate metabolism genes across 107 representative *Butyrivibrio* strains. The top panel shows a phylogenetic tree separating digesta-associated (blue) and epithelium-associated (purple) clades. The bottom heatmap displays the presence (blue) or absence (white) of key enzymes involved in the butyrate pathway (*thl*, *hbd*, *cro*, *buk*, *ptb*, *kamA*, *kce*, *atoA*, *cat2*, *abfD*, *gctA*, *gctB*, *hgdA*, *hgdB*, *gcdA*, and *bcd*). Genes were hierarchically clustered based on presence/absence co-occurrence patterns. Rows correspond to individual genes, and columns represent genomes ordered according to the phylogenetic tree.

**Fig. S9** Genomic locus comparison of *secA* and its neighboring genes in *Butyrivibrio* spp. **A** In digesta-associated clade genomes, the *ptb* (phosphate butyryltransferase) and *buk* (butyrate kinase) genes consistently colocalize with *secA*, which encodes the ATPase motor of the Sec protein translocase system. Each genome harbors a single *secA* gene, and its conserved genomic context with *ptb*/*buk*. **B** In epithelium-associated clade genomes (Completeness > 50%), *secA* is not flanked by *ptb* or *buk*, but instead by genes such as *wecB* (UDP-N-acetylglucosamine 2-epimerase, involved in cell-surface polysaccharide biosynthesis) and accessory Sec components (*asp1–3*, *secY*).

**Fig. S10** Maximum-likelihood phylogenetic tree of nine Campylobacteraceae genomes. The tree includes two previously reported genomes (*Ca*. C. stinkeris and *Ca*. C. noahi) from Strachan et al. (2023) and seven Campylobacteraceae genomes recovered in this study.

**Fig. S11** Quantitative PCR analysis of host DNA across dilution gradients in rumen and colon epithelial samples. Amplification plots of the *Bos taurus* actin beta (*ACTB*) gene are shown for serial dilutions of epithelial cell suspensions ranging from 1:1 to 1:10^6^. **A** Rumen epithelial sample. **B** Colon epithelial sample. The x-axis indicates PCR cycle number, and the y-axis (Rn) represents normalized fluorescence signal intensity. Increasing dilution shifts curves rightward, indicating reduced host genomic DNA. At the highest dilution (1:10^6^), no amplification curve was detected.

**Fig. S12** Validation of negative controls in the Dilute-MetaSeq workflow. Agarose gel electrophoresis of multiple displacement amplification (MDA) reactions. Lanes 1 and 8: 15 kb DNA ladder (L). Lanes 2, 4, 6: no-template MDA reactions with ddH₂O (NTC, negative controls), showing no detectable amplification. Lanes 3, 5, 7: template MDA reactions (T), producing high-molecular-weight DNA products. The absence of products in negative controls (NTC) confirms that MDA reactions were free of detectable contamination.

**Fig. S13** Representative genomic neighborhoods of AMG identified by VIBRANT. Each panel shows a viral contig with its predicted ORFs annotated by functional category. Grey boxes indicate genes without hallmark or AMG annotation. In each case, AMGs are located within genomic regions containing multiple viral hallmark genes, satisfying curation criteria for confident AMG assignment. Gene positions are scaled according to locus length (bp). AMG curation criteria: High-confidence AMGs were retained only if they met all three manual inspection criteria: (i) the AMG was located on a viral contig containing hallmark genes, defined by VOG annotations with keywords such as portal, terminase, spike, capsid, sheath, tail, coat, virion, lysin, holin, base plate, lysozyme, head, or structural; (ii) at least one viral hallmark gene occurred within the ±10 gene neighborhood of the AMG; and (iii) contigs harboring bacterial multi-copy core genes (e.g., 16S rRNA) were excluded.
